# Supplementary material for: Metabolic Features of Increased Gut Permeability, Inflammation, and Altered Energy Metabolism Distinguish Agricultural Workers at Risk for Mesoamerican Nephropathy
Source: Metabolites. 2023 Feb 22;13(3):325. doi: 10.3390/metabo13030325 (PMC10058628; doi:10.3390/metabo13030325)
Supplement: Supplementary file 1 [file metabolites-13-00325-s001.zip › metabolites-2094915-supplementary.pdf]

metabolites

MDPI

Supplementary Table S1. Percent of values for each metabolite below detection limit (BDL) by nuclear magnetic resonance. Percentages are shown overall and separated by occupational group. Metabolites significantly differing in percent of values BDL across occupational groups are shown in bold. CS, can harvest and seed cutter; OW, other cane workers; SW, Spain agricultural workers; NW, non-agricultural workers.

	Overall % BDL *n* = 385	CS % BDL *n* = 117	OW % BDL *n* = 78	SW % BDL *n* = 88	NW % BDL *n* = 102	Chi square *p*-Value
1-Methyladenosine	100%	99%	100%	100%	100%	0.94
1-Methylguanidine	0%	0%	0%	0%	0%	N/A
1-Methylhistidine	85%	77%	90%	83%	91%	0.16
1-Methylhydantoin	0%	0%	0%	0%	0%	N/A
1-Methylnicotinamide	0%	0%	0%	0%	0%	N/A
1,3-Dimethyluric acid	2%	1%	1%	3%	1%	0.92
**2-Furoylglycine**	**63%**	**79%**	**74%**	**35%**	**62%**	**<0.001**
2-Hydroxy-4-methylvaleric acid	92%	93%	96%	90%	88%	0.73
2-Hydroxyisovaleric acid	22%	22%	27%	19%	22%	0.98
2-Hydroxyphenylacetic acid	83%	78%	95%	85%	79%	0.12
2-Ketobutyric acid	98%	99%	99%	98%	98%	>0.99
2-Methylsuccinic acid	96%	94%	97%	98%	94%	0.90
2-Oxoglutaric acid	28%	35%	35%	17%	23%	0.12
2-Oxoisocaproic acid	57%	61%	51%	60%	54%	0.93
2-Oxoisovaleric acid	97%	98%	99%	99%	93%	0.32
3-Aminoisobutyric acid	78%	78%	76%	82%	75%	0.99
3-Hydroxy-3-methylglutaric acid	90%	88%	95%	89%	90%	0.91
3-Hydroxybutyric acid	55%	53%	65%	48%	56%	0.60
3-Hydroxyglutaric acid	28%	29%	35%	17%	29%	0.42
3-Hydroxyisovaleric acid	0%	0%	0%	0%	0%	N/A
3-Hydroxypropionic acid	70%	75%	68%	68%	69%	0.97
3-Hydroxyvaleric acid	99%	99%	100%	99%	97%	0.85
3-Methyl-2-oxovaleric acid	36%	39%	29%	44%	31%	0.59
3-Methylcrotonylglycine	96%	96%	92%	99%	96%	0.72
3-Methylglutaconic acid	1%	1%	1%	0%	0%	0.95
3-Phenyllactic acid	51%	49%	54%	44%	57%	0.84
4-Aminobutyric acid	64%	69%	64%	57%	65%	0.85
4-Aminohippuric acid	100%	100%	100%	100%	100%	N/A
4-Ethylphenol	100%	100%	100%	100%	99%	0.90
**4-Hydroxyhippuric acid**	**85%**	**92%**	**92%**	**78%**	**77%**	**0.03**
4-Hydroxyphenylacetic acid	41%	35%	46%	42%	43%	0.90
4-Hydroxyphenyllactic acid	95%	97%	91%	94%	95%	0.78
4-Hydroxyphenylpyruvic acid	81%	86%	85%	76%	75%	0.45
4-Pyridoxic acid	6%	5%	9%	2%	7%	0.80
5-Aminolevulinic acid	98%	98%	99%	98%	97%	>0.99
5-Aminopentanoic acid	100%	100%	100%	100%	100%	N/A

Acetaminophen glucuronide	97%	97%	99%	99%	95%	0.86
Acetic acid	0%	0%	0%	0%	0%	N/A
Acetoacetic acid	7%	3%	8%	15%	4%	0.06
Acetoine	100%	100%	100%	100%	100%	N/A
Acetone	1%	0%	0%	0%	2%	0.59
Adenine	8%	9%	10%	7%	7%	>0.99
Adenosine	100%	100%	100%	100%	100%	N/A
Alanine	0%	0%	0%	0%	0%	N/A
Allantoin	1%	2%	0%	0%	1%	0.91
Arginine	7%	9%	6%	8%	6%	>0.99
Argininosuccinic acid	99%	98%	100%	100%	99%	0.91
Benzoic acid	97%	98%	97%	92%	98%	0.39
Betaine	2%	2%	3%	1%	1%	>0.99
Butyric acid	79%	79%	82%	75%	80%	0.98
Caffeine	0%	0%	0%	0%	0%	N/A
Choline	73%	74%	60%	81%	74%	0.24
Citraconic acid	100%	100%	100%	100%	99%	0.90
Citric acid	0%	0%	0%	0%	0%	N/A
Citrulline	35%	32%	36%	47%	28%	0.38
**Creatine**	**15%**	**32%**	**8%**	**7%**	**7%**	**<0.001**
Cystine	97%	97%	99%	97%	97%	>0.99
Cytosine	67%	70%	78%	56%	65%	0.17
D-Galactonic acid	90%	88%	86%	90%	97%	0.35
D-Galactose	69%	68%	72%	64%	73%	0.95
D-Gluconic acid	82%	88%	82%	76%	79%	0.62
D-Glucose	1%	1%	0%	0%	1%	0.98
**D-Mannitol**	**95%**	**98%**	**95%**	**86%**	**98%**	**0.01**
D-Mannose	77%	87%	78%	72%	71%	0.15
D-Panthenol	100%	100%	99%	100%	100%	0.79
Dihydrothymine	4%	8%	3%	1%	4%	0.52
Dihydrouracil	72%	73%	71%	77%	69%	0.97
Dimethylamine	0%	0%	0%	0%	0%	N/A
Ethanol	96%	97%	92%	94%	97%	0.80
Ethylmalonic acid	12%	14%	18%	6%	10%	0.45
Formic acid	0%	0%	0%	0%	0%	N/A
Fumaric acid	0%	0%	0%	0%	0%	N/A
Galactitol	96%	99%	96%	92%	94%	0.46
Glutaconic acid	100%	100%	100%	100%	100%	N/A
Glutamic acid	99%	99%	100%	99%	97%	0.85
Glutamine	93%	92%	88%	97%	95%	0.65
Glutaric acid	37%	34%	42%	30%	42%	0.71
Glycerol	99%	98%	100%	100%	100%	0.71
Glycine	0%	0%	0%	0%	0%	N/A
Glycolic acid	0%	0%	0%	0%	0%	N/A

Guanidinoacetic acid	0%	0%	0%	0%	0%	N/A
Hippuric acid	0%	0%	0%	0%	0%	N/A
Imidazole	34%	31%	31%	42%	34%	0.84
Inosine	2%	3%	1%	0%	2%	0.94
Isobutyrylglycine	100%	100%	100%	100%	100%	N/A
Isopropanol	99%	99%	100%	100%	99%	0.98
Kynurenate	0%	0%	0%	0%	0%	N/A
**L-Alloisoleucine**	**80%**	**74%**	**68%**	**93%**	**84%**	**<0.01**
L-Ascorbic acid	100%	100%	100%	100%	100%	N/A
L-Carnosine	100%	100%	100%	100%	100%	N/A
L-Citramalic acid	1%	1%	0%	1%	1%	>0.99
L-Fucose	13%	14%	10%	13%	14%	>0.99
L-Homocystine	100%	100%	100%	100%	100%	N/A
L-Isoleucine	23%	26%	21%	15%	29%	0.49
L-Kynurenine	100%	100%	100%	100%	100%	N/A
L-Pyroglutamic acid	75%	74%	69%	83%	74%	0.71
L-Tryptophan	10%	14%	8%	8%	9%	0.90
L-Tyrosine	100%	100%	100%	100%	100%	N/A
Lactic acid	1%	1%	1%	0%	0%	0.95
Lactose	4%	5%	6%	1%	2%	0.68
Leucine	8%	13%	10%	1%	7%	0.19
Maleic acid	3%	3%	5%	0%	3%	0.74
Malic acid	100%	100%	100%	99%	100%	0.85
Mandelic acid	90%	90%	94%	92%	84%	0.66
Methanol	1%	2%	0%	1%	0%	0.90
Methionine	63%	57%	64%	69%	63%	0.87
**Methylmalonic acid**	**62%**	**62%**	**71%**	**40%**	**75%**	**<0.001**
Myo-Inositol	95%	92%	97%	95%	97%	0.80
N-Acetylaspartic acid	0%	0%	0%	0%	0%	N/A
N-Acetylglutamate	90%	91%	85%	84%	97%	0.11
N-Acetylphenylalanine	97%	96%	99%	95%	97%	0.97
N-Acetyltyrosine	100%	100%	100%	100%	100%	N/A
N-Isovaleroylglycine	35%	32%	38%	40%	30%	0.92
N,N-Dimethylglycine	0%	0%	0%	0%	0%	N/A
NAD+	0%	0%	0%	0%	0%	N/A
Neopterin	0%	0%	0%	0%	0%	N/A
Nicotinamide	0%	0%	0%	0%	0%	N/A
Orotic acid	0%	0%	0%	0%	0%	N/A
Oxaloacetic acid	3%	3%	1%	5%	2%	0.96
Pantothenic acid	0%	0%	0%	0%	0%	N/A
Paracetamol	90%	87%	92%	92%	89%	0.96
Phenylacetic acid	11%	17%	12%	10%	6%	0.44
Phenylalanine	16%	18%	10%	17%	16%	0.94
Phenylpyruvic acid	12%	13%	8%	17%	12%	0.85

Pimelic acid	98%	98%	96%	99%	99%	0.93
Proline betaine	3%	2%	1%	6%	2%	0.74
Propionic acid	29%	28%	31%	34%	25%	0.97
Propionylglycine	99%	99%	100%	99%	100%	0.97
Propylene glycol	14%	8%	9%	19%	19%	0.21
Pyrocatechol	100%	100%	100%	100%	100%	N/A
Pyruvic acid	0%	0%	0%	0%	0%	N/A
Quinolinic acid	100%	100%	100%	100%	100%	N/A
Sarcosine	19%	19%	19%	16%	24%	0.97
Succinic acid	0%	0%	0%	0%	0%	N/A
Succinylacetone	93%	95%	96%	90%	91%	0.81
Syringic acid	0%	0%	0%	0%	0%	N/A
Tartaric acid	1%	0%	0%	0%	2%	0.59
Taurine	2%	3%	0%	5%	2%	0.80
Theobromine	0%	0%	0%	0%	0%	N/A
Threonic acid	98%	97%	100%	99%	95%	0.61
Thymine	75%	74%	71%	76%	78%	0.98
**Thymol**	**11%**	**16%**	**15%**	**0%**	**12%**	**0.03**
**Tiglylglycine**	**88%**	**92%**	**92%**	**73%**	**93%**	**<0.001**
Trigonelline	0%	0%	0%	0%	0%	N/A
Trimethylamine	0%	0%	0%	0%	0%	N/A
Tyramine	96%	97%	95%	91%	99%	0.26
Uracil	17%	24%	18%	10%	15%	0.41
**Uridine**	**27%**	**36%**	**27%**	**10%**	**30%**	**0.01**
Valine	2%	3%	4%	0%	0%	0.43
Xanthurenic acid	22%	23%	29%	16%	19%	0.63

Supplementary Table S2. Key metabolites of NAD+, tryptophan, and nicotinamide metabolism, with cane harvest and seed cutters separated. Groups hypothesized to be at greater risk of metabolic derangement along these metabolic pathways are orange, at less risk blue. * indicates a statistically significant difference from the cane cutters group at a p-value of <0.05, ** at <0.01. NAD+, nicotinamide adenine dinucleotide +, IQR, interquartile range.

	Cane Harvest Cutters (n=82)	Cane Seed Cutters (n=35)	Other Cane Workers (n=78)	Spain Agricultural Workers (n=78)	Non-Agricultural Workers [illegible]	p
**NAD+**						
median	0.0155	0.0162	0.0126**	0.0122*	0.0146	0.001
IQR	0.0120, 0.0197	0.0117, 0.0204	0.0094, 0.0164	0.0093, 0.0181	0.0124, 0.0185	
**Tryptophan**						
median	0.0054	0.0067	0.0055	0.0061	0.0068**	0.01
IQR	0.0033, 0.0067	0.0039, 0.0079	0.0042, 0.0075	0.0043, 0.0094	0.0043, 0.0093	
**Kynurenic acid**						
median	0.0200	0.0203	0.0160**	0.0184	0.0188	<0.0001
IQR	0.0166, 0.0232	0.0165, 0.0263	0.0140, 0.0189	0.0160, 0.0255	0.0152, 0.0217	
**Nicotinamide**						
median	0.0144	0.0137	0.0130	0.0139	0.0126	0.34
IQR	0.0112, 0.0184	0.0109, 0.0166	0.0112, 0.0182	0.0104, 0.0260	0.0101, 0.0167	
**Methylnicotinamide**						
median	0.0040	0.0036	0.0039	0.0035	0.0048	0.0008
IQR	0.0030, 0.0056	0.0026, 0.0054	0.0031, 0.0057	0.0027, 0.0047	0.0034, 0.0069	
**Hippurate**						
median	0.695	0.869	0.148**	0.166**	0.176**	<0.0001
IQR	0.395, 1.254	0.380, 1.330	0.088, 0.256	0.088, 0.256	0.117, 0.259	
**KYNURENINE PATHWAY**						
**NAD+ to Tryptophan Ratio**						
median	3.15	2.68	2.32	2.12*	2.06*	0.01
IQR	2.00, 6.29	1.96, 5.08	1.53, 4.24	1.32, 4.89	1.43, 4.49	
**Kynurenic Acid to Tryptophan Ratio**						
median	3.60	3.26	2.89*	3.06	2.66**	0.002
IQR	2.71, 6.40	2.40, 5.67	2.12, 3.91	2.15, 4.88	1.95, 4.19	
**NAD+ to Kynurenic Acid Ratio**						
median	0.700	0.752	0.698	0.594	0.758	0.03
IQR	0.542, 1.083	0.517, 1.055	0.561, 0.896	0.462, 1.138	0.610, 0.990	
**SALVAGE PATHWAY**						
**NAD+ to Nicotinamide Ratio**						
median	0.985	1.072	0.939	0.924	1.193	0.13

IQR	0.776, 1.616	0.742, 1.587	0.684, 1.260	0.351, 1.796	0.773, 1.627	
**Methylnicotinamide to Nicotinamide Ratio**						
median	0.323	0.280	0.301	0.236*	0.374	<0.0001
IQR	0.217, 0.427	0.202, 0.331	0.193, 0.500	0.110, 0.346	0.248, 0.575	
**NAD+ to Methylnicotinamide Ratio**						
median	3.68	3.35	2.72*	3.52	3.01	0.02
IQR	2.78, 5.64	2.63, 6.81	2.17, 3.93	2.47, 5.31	2.07, 4.90	

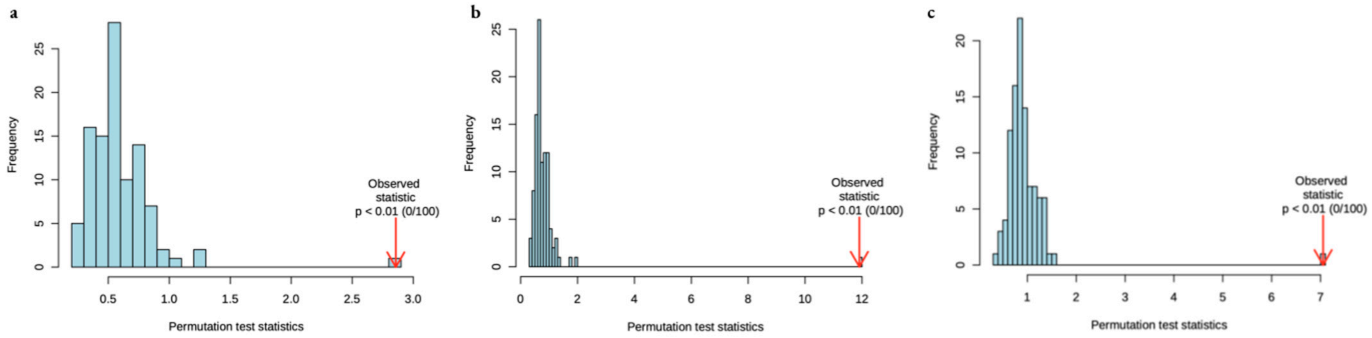

Supplementary Figure S1. Partial Least Squares Discriminant Analysis (PLS-DA) model validation by permutation tests based on separation distance. Validation is shown for PLS-DA models comparing Nicaraguan sugarcane harvest and seed cutters (CS) with (a) other Nicaraguan sugarcane workers (OW), (b) agricultural workers in Spain (SW), and (c) non-agricultural workers in Nicaragua (NW).

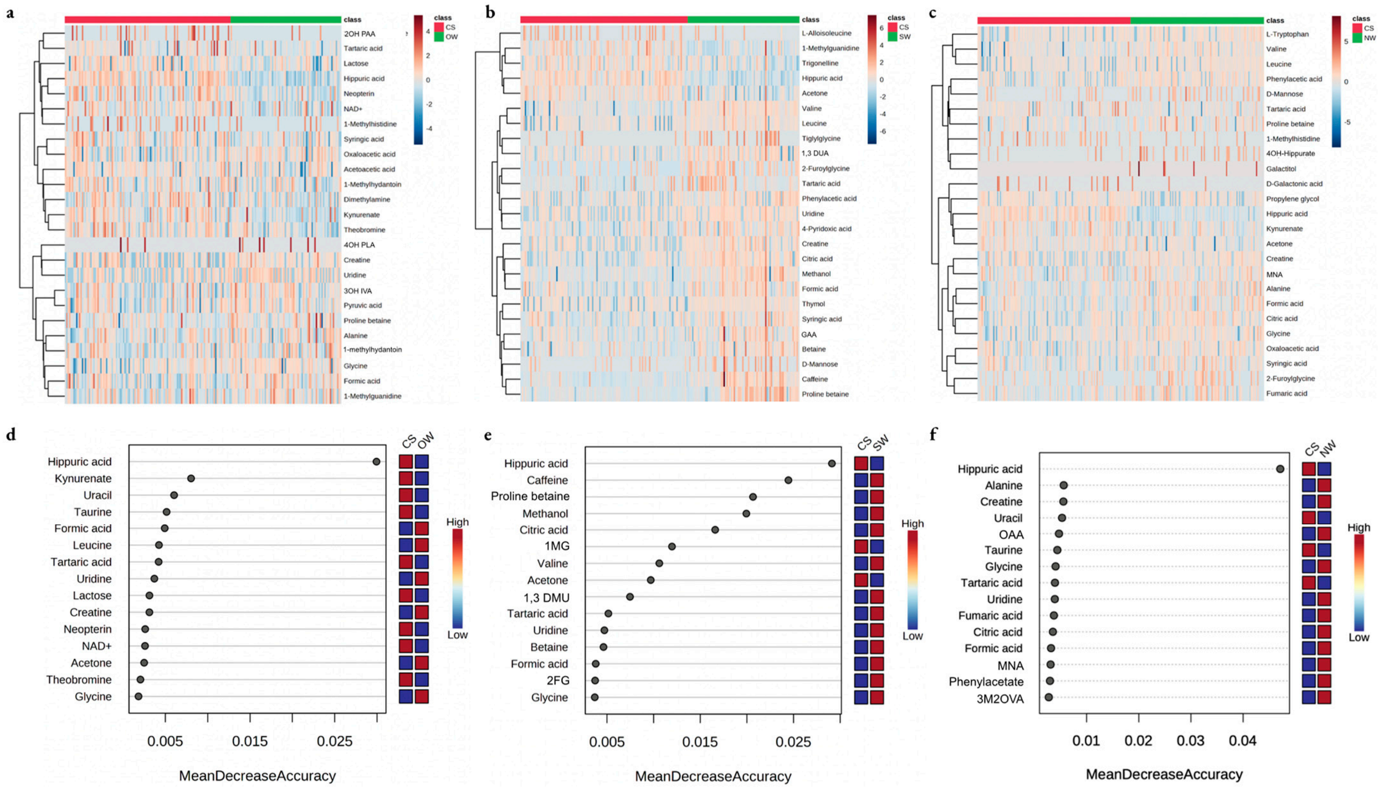

Supplementary Figure S2. Differential metabolic features of urine between labor-based comparison groups. Heatmap of the top 25 metabolic features by t-test comparing Nicaraguan sugarcane harvest and seed cutters with (a) other Nicaraguan sugarcane workers, (b) agricultural workers in Spain, and (c) non-agricultural workers in Nicaragua. Important features by mean decrease in accuracy score in random forest models are shown in Nicaraguan sugarcane harvest and seed cutters compared to (d) other Nicaraguan sugarcane workers, (e) agricultural workers in Spain, and (f) non-agricultural workers in Nicaragua. 3OH IVA, 3-hydroxyisovaleric acid; 2OH PAA, 2-hydroxyphenylacetic acid; 4OH PLA, 4-hydroxyphenyllactic acid; NAD+, nicotinamide adenine dinucleotide; 1,3 DUA, 1,3 dimethyluric acid; GAA, guanidinoacetic acid; OAA, oxaloacetic acid; 1MG, 1-methylguanidine; 1,3 DMU, 1,3 dimethyluracil; 2FG, 2-furoylglycine; MNA, 1-methylnicotinamide; 3M2OVA, 3-methyl-2-oxovaleric acid.

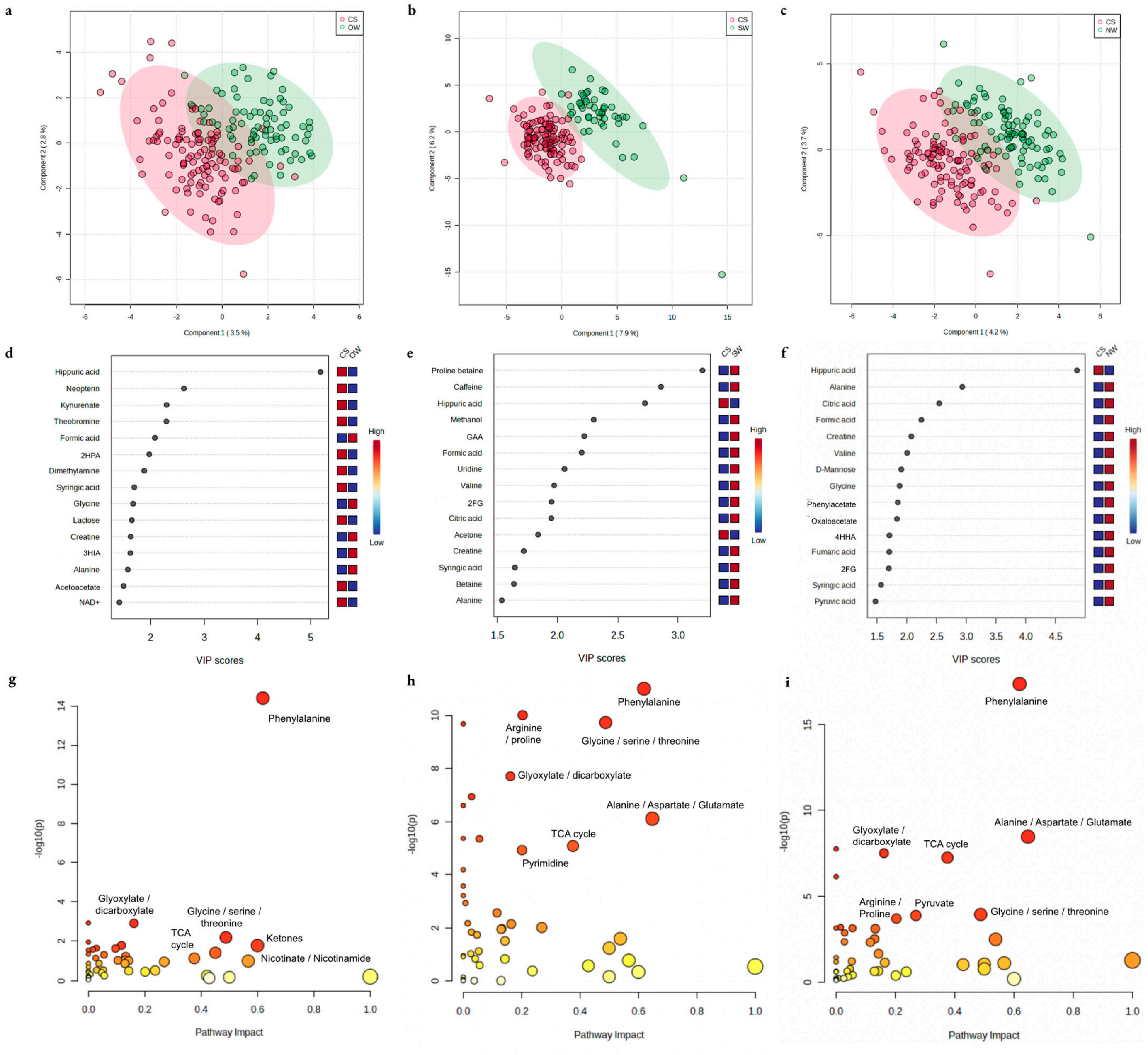

Supplementary Figure S3. Differential metabolic features of urine between comparison groups using partial least squares discriminant analysis (PLS-DA), with analysis restricted to the subset of participants with an eGFR of 90 ml/min/1.73m^2 or greater. 2D visualization of separation of groups by PLS-DA is shown in Nicaraguan Cane Harvest and Seed Cutters (CS) compared with (a) Other Cane Workers (OW) in Nicaragua, (b) Spain Agricultural Workers (SW), and (c) Non-Agricultural Workers (NW) in Nicaragua. Important features by variable importance in prognosis (VIP) score in PLS-DA are shown in the CS group compared to the (d) OW, (e) SW, and (f) NW groups. Significant metabolic pathways differentiating groups are shown in the CS group compared to the (g) OW, (h) SW, and (i) NW groups. 2HPA, 2-hydroxyphenylacetic acid; 3HIA, 3-hydroxyisovaleric acid; NAD+, nicotinamide adenine dinucleotide; 2FG, 2-furoylglycine; 1MG, 1-methylguanidine; GAA, guanidinoacetic acid; 4HHA, 4-hydroxyhippuric acid TCA, tricarboxylic acid.
